# Liver elasticity in healthy individuals by two novel shear-wave elastography systems—Comparison by age, gender, BMI and number of measurements

**DOI:** 10.1371/journal.pone.0203486

**Published:** 2018-09-14

**Authors:** Anesa Mulabecirovic, Anders Batman Mjelle, Odd Helge Gilja, Mette Vesterhus, Roald Flesland Havre

**Affiliations:** 1 Department of Clinical Medicine, University of Bergen, Bergen, Norway; 2 National Centre for Ultrasound in Gastroenterology, Haukeland University Hospital, Bergen, Norway; 3 Norwegian PSC Research Center, Department of Transplantation Medicine, Division of Cancer Medicine, Surgery, Inflammatory Diseases and Transplantation, Oslo University Hospital, Oslo, Norway; Medizinische Fakultat der RWTH Aachen, GERMANY

## Abstract

**Objective:**

Establishing normal liver stiffness (LS) values in healthy livers is a prerequisite to differentiate normal from pathological LS values. Our aim was to define normal LS using two novel elastography methods head-to-head and to assess the number of measurements, variability and reproducibility.

**Materials and methods:**

We evaluated shear wave elastography (SWE) methods integrated in Samsung RS80A and GE S8 by obtaining LS measurements (LSM) in 100 healthy subjects (20–70 years). Transient Elastography (TE) was used as reference method. Data were analyzed according to age, sex, BMI and 5 vs. 10 measurements. All subjects underwent B-mode ultrasound examination and lab tests to exclude liver pathology. Interobserver variation was evaluated in a subset (n = 24).

**Results:**

Both methods showed excellent feasibility, measuring LS in all subjects. LSM-mean for GE S8 2D-SWE was higher compared to TE (4.5±0.8 kPa vs. 4.2±1.1, p<0.001) and Samsung RS80A (4.1±0.8 kPa, p<0.001). Both methods showed low intra- and interobserver variation. LSM-mean was significantly higher in males than females using 2D-SWE, while a similar trend for Samsung SWE did not reach significance. No method demonstrated statistical significant difference in LSM across age and BMI groups nor between LSM-mean based on 5 vs. 10 measurements.

**Conclusion:**

LSM was performed with high reproducibility in healthy adult livers. LSM-mean was significantly higher for GE S8 2D-SWE compared to Samsung RS80A and TE in healthy livers. Males had higher LSM than females. No method demonstrated statistical significant difference in LSM-mean across age- and non-obese BMI groups. Our results indicate that five LSM may be sufficient for reliable results.

## Introduction

Chronic liver disease is one of the leading causes of morbidity and mortality worldwide [1, 2]. Assessment of liver fibrosis is important for chronic liver disease of various aetiologies for outcome prediction, risk stratification and selection for screening programs (e.g. endoscopy for oesophageal varices) as well as therapeutic decisions [2]. Non-invasive methods including ultrasound elastography have emerged within the past decade and are increasingly replacing liver biopsy for liver fibrosis assessment, avoiding the risks and discomforts of this invasive method. Nonetheless, ultrasound elastography encompasses several methods with important technological differences, ranging from vibration-controlled transient elastography (TE, Fibroscan) to methods based on deposition of an acoustic pulse such as point shear wave elastography (pSWE) and more recently 2D-SWE. TE has been extensively validated and is recommended for clinical use by several international guidelines, and an increasing number of studies evaluating the accuracy of various elastography methods have provided evidence for the utility of elastography imaging. However, with the expanding spectrum of ultrasound based elastography systems, it has become increasingly clear that the various technologies and platforms may yield different estimates of liver stiffness (LS) within the same liver. Hence, current guidelines acknowledge a need to establish reference values for normal liver stiffness in healthy livers for each specific equipment model in order to allow accurate diagnosis of pathological liver stiffness[3, 4].

To our knowledge, this is the first study to evaluate liver stiffness measurements (LSM) in healthy liver subjects using 2D-SWE from GE Logiq S8 (GE Healthcare, Milwaukee, Wi, USA) as well as SWE from Samsung RS80A (Samsung Medical, Seoul, Korea). Our study primarily aimed to define normal values of liver stiffness (LS) for males and females across adult age groups using these two novel platforms. We applied TE using Fibroscan integrated in the GE Logiq S8 ultrasound scanner (Echosens, Paris, France) as a reference method. Furthermore, we aimed to analyse influencing factors, such as BMI, and to assess the inter- and intraobserver variability and reproducibility, as well as to investigate the difference between obtaining five and ten consecutive liver stiffness measurements in order to calculate a representative median liver stiffness measurement (LSM).

## Material and methods

### Study design and subject population

The study was designed as a single-centre cross-sectional prospective study in selected healthy individuals. The protocol was in accordance with the Declaration of Helsinki for research in medicine and biology, and was approved by the Regional Committee for Medical and Health Research Ethics in Western Norway. All subjects were given oral and written information about the study and were invited to participate. Informed written consent was obtained from each subject enrolled. The study was performed in August and September 2017, at the Department of Gastroenterology, Haukeland University Hospital in Bergen, Norway.

**Fig 1:**
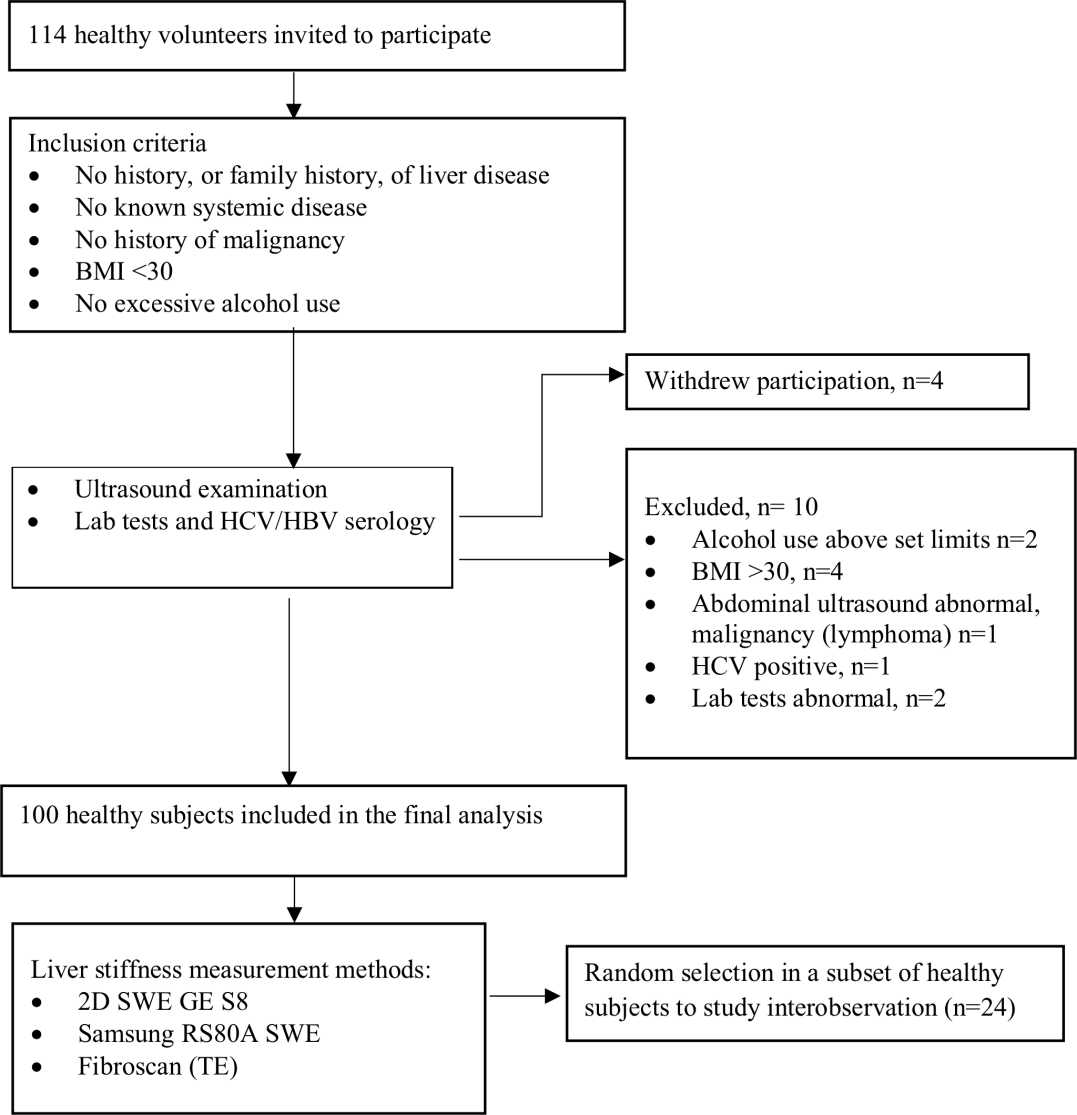
Method of selection of healthy subjects. Flow chart of data collection.

The characteristics of healthy subjects are shown in Table 1. The subjects consisted of volunteers with various occupational backgrounds recruited amongst staff, their families and social network. Volunteers were recruited into five groups by age, with 10 males and 10 females per group: 20–30, 31–40, 41–50, 51–60 and 61–70 years (Table 1). Liver disease was ruled out as far as possible by patients’ history, laboratory tests and negative viral markers. In total ten subjects were excluded, weekly alcohol use extending 10 units for males and 6 units for females (n = 2), abnormal laboratory tests (n = 3) or evidence of malignancy on ultrasound examination (n = 1). Individuals with BMI >30 kg/m^2^ were excluded (n = 4). Four subjects withdrew their participation consent (Fig 1). We included 100 healthy subjects in the final analysis. A random subset of subjects (n = 24) were included for assessment of interobserver variability. For analyses regarding the effect of BMI, the subjects were divided into two groups with BMI between 18.0 and 25 kg/m^2^ (n = 73) and BMI between 25 and 30 kg/m^2^ (n = 27), respectively.

**Table 1:** The characteristics of healthy subjects, by age group.

	20–30 (n = 20)	31–40 (n = 20)	41–50 (n = 20)	51–60 (n = 20)	61–70 (n = 20)
***Characteristics***					
Age, years^⊥^ (range)	27.8 ± 2 (25–30)	34.1 ± 2.7 (31–40)	44.3 ± 3 (41–50)	55.7 ± 3 (51–60)	64.15 ± 2.5 (61–69)
Gender; Female/Male, n	10/10	10/10	10/10	10/10	10/10
BMI, kg/m^2⊥^ (range)	22.5 ± 2.4 (19.4–27.2)	23.8 ± 2.3 (20.7–28.4)	24.4 ± 3 (18.1–28.7)	24.5 ± 2.3 (20–29.9)	24.7 ± 2.9 (20–29.6)
Weight group^⊥^, n (%)					
18.0–25 kg/m^2^	17 (17%)	15 (15%)	15 (15%)	13 (13%)	13 (13%)
25.0–30 kg/m^2^	3 (3%)	5 (5%)	5 (5%)	7 (7%)	7 (7%)
Alcohol units per week^⊥^(range)	4.4 ± 2.5 (0–8)	3.8 ± 2 (0–6)	3.7 ± 2.6 (1–10)	4.4 ± 2.6 (0–10)	4.2 ± 3 (0–10)
***Biochemical profile***					
Total bilirubin μmol/L* [<19 μmol/L] *(IQR; range)*	10.5 (8.7–12.5; 4–18)	17 (5.5–15.3; 3–39)	9.5 (7.5–11.4; 4–21)	8.5 (7.1–12.1; 4–28)	8.6 (7.4–9.7; 4–15)
AST, U/L * [15–45 U/L] *(IQR; range)*	23.5 (21.9–28.6; 17–41)	25.0 (21.6–27.7; 15–45)	22.0 (20.5–26.1; 15–40)	23.0 (21.7–25.7; 16–32)	23.5 (21.5–27.8; 13–37)
ALT, U/L * [10–70 U/L] *(IQR; range)*	17.0 (16.9–28.4; 11–55)	22.5 (18.7–27.1; 8–45)	23.0 (21.5–30.7; 15–47)	22.5 (20.1–30.7; 14–40)	24.5 (22–28.7; 14–46)
GGT, U/L* [10–115 U/L] *(IQR; range)*	18.5 (15.5–22.1; 7–42)	15.0 (13.5–19.5; 9–29)	19.0 (17.0–34.4; 5–68)	17.0 (16.7–26.0; 11–48)	22.5 (17.0–35.3; 9–96)
Serum Albumin, g/L* [39–50 g/L] *(IQR; range)*	48.0 (46.4–49.3; 41–53)	47.0 (45.6–48.2; 43–52)	46.5 (45.2–47.4; 41–51)	46.5 (45.2–49.7; 43–66)	46.0 (45.1–46.8; 43–50)
Platelet counts, 10^9^ /L* [145–387 10^9^ /L] *(IQR; range)*	237.5 (210.7–255.5; 152–352)	222.5 (210.6–254.8; 155–334)	256.5 (229.9–284.4; 141–400)	244.5 (221.8–273.8; 144–355)	245.5 (221.3–271.8; 165–365)
APRI score* *(IQR; range)*	0.29 (0.26–0.37; 0.17–0.56)	0.29 (0.26–0.37; 0.15–0.68)	0.26 (0.22–0.31; 0.13–0.45)	0.26 (0.24–0.34; 0.18–0.58)	0.31 (0.25–0.35; 0.10–0.55)
FIB-4 score* *(IQR; range)*	0.66 (0.59–0.77; 0.4–1.09)	0.79 (0.72–0.93; 0.42–1.41)	0.82 (0.73–1.09; 0.42–1.93)	1.12 (0.99–1.36; 0.61–2.01)	1.38 (1.05–1.56; 0.52–2.38)

*Data are presented as median ⊥Data are presented as mean ± SD. SD, Standard deviation; IQR, Interquartile range (representing upper and lower bound); Range (from minimum value to maximum value). BMI, Body Mass Index; ALT, alanine aminotransferase; AST, aspartate aminotransferase; APRI, AST to Platelet Ratio Index; FIB4, Fibrosis-4. Reference values for our laboratory tests are given in the brackets, normal values cover both genders.

### Laboratory analyses

On the day of ultrasound and elastography, blood was sampled and biochemical analyses were performed using standard routine laboratory protocols. The tests included C-reactive protein (CRP), haemoglobin, leukocytes, platelets, creatinine, total bilirubin, albumin, international normalization rate (INR), aspartate aminotransferase (AST), alanine aminotransferase (ALT), alkaline phosphatase (ALP) and gamma-glutamyl transferase (GGT). The laboratory analyses were performed in our hospital’s laboratory, and reference values were gender specific. Three subjects had a bilirubin value outside the gender specific reference range, but none of these were excluded as the values normalized and diagnostic work-up showed no evidence of liver disease. Viral markers for hepatitis C virus (HCV) and hepatitis B virus (HBV) were also included. APRI and FIB-4 scores of fibrosis were calculated using published algorithms [5, 6].

### B-mode ultrasound examination

All subjects underwent B-mode ultrasound examination of the liver, gallbladder, spleen and kidneys using a Samsung RS80A before SWE examination. All examinations were conducted after a minimum of four hours of fasting, using a standardized scanning protocol and by a single operator (AM) with >3 years’ experience in abdominal ultrasound. Small hepatic capillary haemangiomas were found in 9 subjects; none of these subjects were excluded as the lesions were confirmed by contrast enhanced ultrasound and were considered small and unlikely to influence the liver stiffness.

### Elastography methods and SWE examination

Three shear wave elastography (SWE) methods were assessed in the study and are listed below in chronological order of assessment. The scanner settings were standardized for all systems. All measurements were performed by a single operator (A.M.). In order to evaluate interobserver variation, a subset of subjects (n = 24) were examined by two independent observers (A.M. and A.B.M.). Observer A (A.M.) and B (A.B.M.) had >3 and 1 years’ experience in ultrasound liver scanning and elastography, respectively. The subjects were fasting (minimum 4 hours) and examined in the supine position with their right arm abducted. All SWE measurements were obtained in the right liver lobe through an intercostal space in relaxed mid-breath hold with minimal transducer pressure being applied; for Samsung RS80A and GE S8 the measurements were acquired in the right lobe about 2 cm beneath the Glisson capsule, perpendicular to the capsule, avoiding large liver vessels, bile ducts and rib shadow in B-mode. Each observer performed first 10, and then 5 separate measurements in the same area with each of the ultrasound based elastography methods. A valid LS assessment was considered as the median value and range of 10 and 5 measurements, acquired in a homogenous area (Samsung RS80A) or in a homogenous elastogram (GE S8 2D-SWE) with an interquartile range (IQR)/median <30% and a success rate (SR) ≥60%.

#### Samsung RS80A SWE

**Fig 2:**
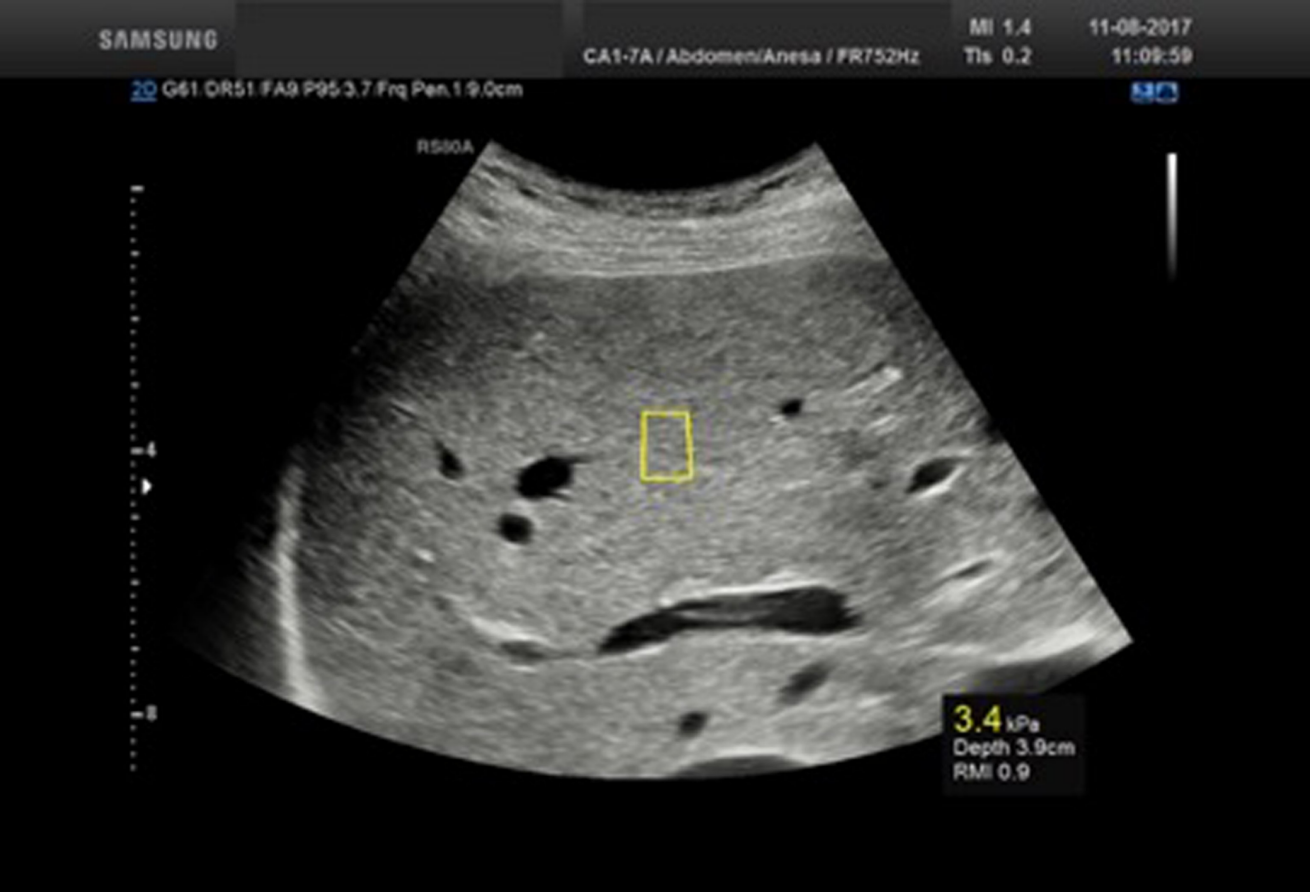
Samsung RS80A SWE performed on a healthy liver. The figure illustrates Samsung RS80A SWE method performed on a healthy subject. The yellow box (centre) represents the shear-wave measurement area and is expressed below the obtained elasticity measurement of 3.4 kPa.

The Prestige ultrasound system (Samsung Medison Co. Ltd., Seoul, Korea) was applied using a CA1-7A convex array probe with a frequency of 1–7 MHz. The software version was 3.00.03.0824. The method measured the average liver elasticity within a region of interest (ROI). Within the brightness mode (B-mode) window, using default scanner settings, the ROI could be placed freely, with a fixed height of 10 mm. The width was automatically adjusted depending on the measurement depth (Fig 2). LSM was expressed in kilopascals (kPa) and meters per second (m/s).

#### GE Logiq S8 2D-SWE

**Fig 3:**
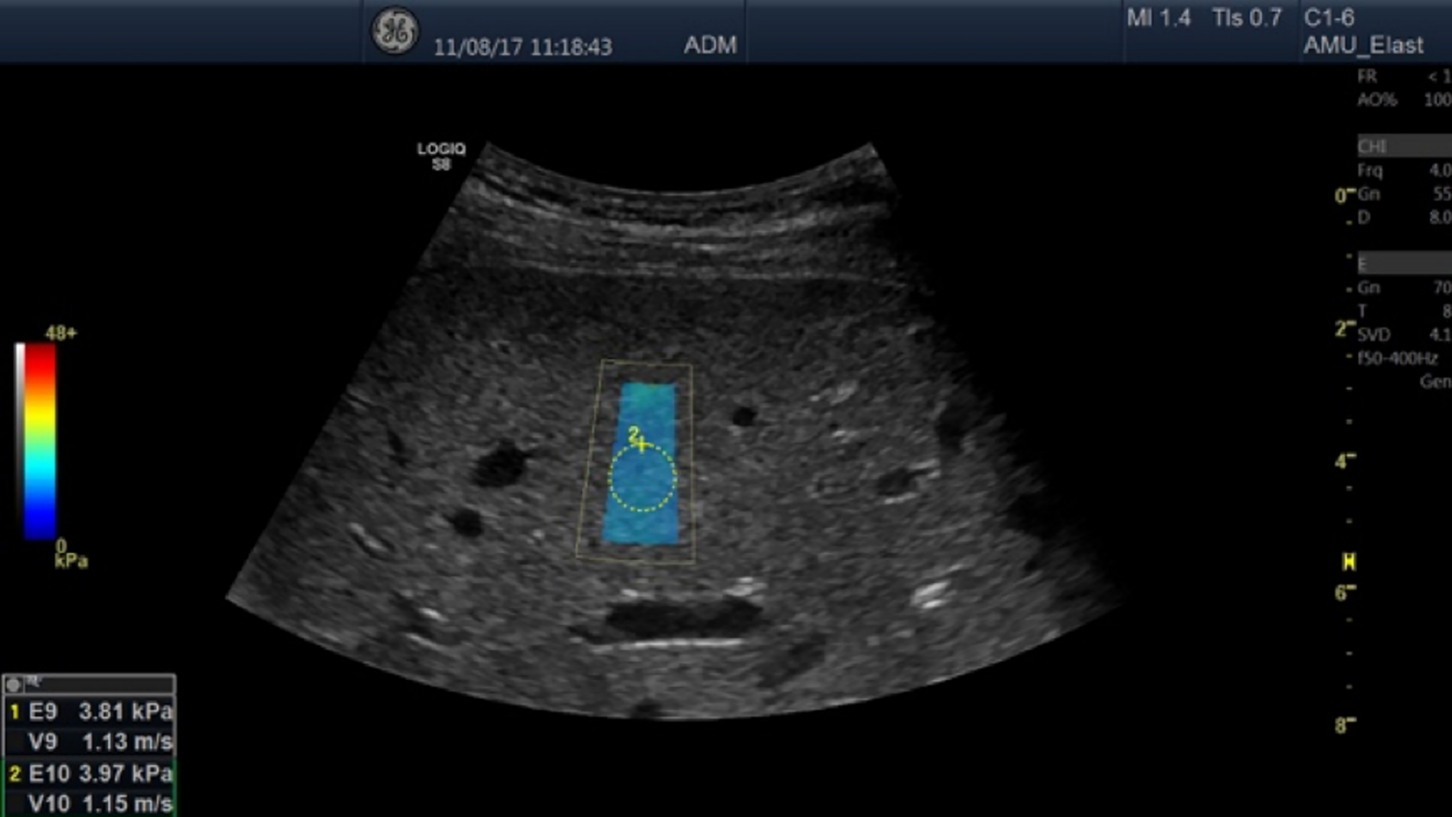
2D-SWE by GE S8 performed on a healthy liver. The figure illustrates the method of 2D-SWE by GE performed on a healthy subject. The coloured box (centre) represents the elastogram, and the circle represents the ROI where the elastic modulus (LSM, liver stiffness measurement) of the liver is acquired. The blue colour indicates soft liver tissue, as semi-quantitatively presented by the colour scale to the left.

2D-SWE from the S8 Ultrasound scanner (GE Healthcare, Milwaukee, Wisconsin, USA), Version R4.1.2, was applied using the C1-6 convex array probe with a frequency of 1–6 MHz. Within the elastogram a circular ROI was placed, standardized to 10 mm in our study and under default scanner settings. The elastic modulus of the liver was automatically acquired by the system. The colour 2D-SWE images were captured and 2–3 elasticity frames per breath-hold (3–5 seconds) were recorded. One ROI was placed within each homogenously coloured elastogram (Fig 3). LSM was expressed in m/s and kPa.

#### Transient elastography (TE)

Integrated in the GE Logiq S8 ultrasound scanner, TE (Fibroscan®, EchoSens, Paris, France), was applied using the M-probe with a frequency of 3.5 MHz and used according to the manufacturer’s instructions. A reliable and valid measurement acquisition was defined as SR ≥60% and IQR/median <30% [7].

### Statistical analysis

The statistical analysis was performed using SPSS, Version 24.0, IBM Statistics (Armon, New York, NY, USA). We used descriptive statistics for demographic, clinical and laboratory characteristics. Sample size power estimation was performed using a 2-sided comparison of two-means model. Estimating a difference in means of 4.0–4.5 kPa with a standard deviation of 0.5 kPa between the methods, 80% power and type I error of 5% yielded a sample size of 16; we compared groups consisting of 20 individuals or more. Variables were tested for normal distribution by calculation and graphics using the Shapiro Wilk test and Q-Q Plot. Differences between numerical variables with a normal distribution were assessed with parametric tests (t-test), and those with a non-normal distribution, with nonparametric tests (Mann-Whitney). P-values of < 0.05 were considered significant. Data are presented as mean (SD) when the data were normally distributed. We calculated the coefficient of variation (CV) of the intraobserver variability. Inter-class correlation coefficients (ICC) were calculated to present the interobserver reliability. Inter-observer agreement was classified as poor (0.00–0.20), fair (0.21–0.40), moderate (0.41–0.60), good (0.61–0.80) and excellent (0.81–1.00) [8]. Correlations were tested by Pearson correlation coefficient. Limits of agreement were assessed according to Bland and Altman to discover differences between individual measurements and to detect possible biases for each method [9, 10]. IQR/Median (%) was calculated for both observers individually as well as together, and for all systems [3, 11].

## Results

A total of 100 healthy subjects were included. LSM was obtained by three different elastography methods (Samsung RS80A, GE S8 2D-SWE and TE). The feasibility of the methods was excellent and successful measurements were obtained in all 100 subjects by all three methods. The characteristics of the healthy subjects are shown in Table 1.

### Measurement variability for the different elastography methods

The overall mean value of the median liver stiffness (LSM-mean) in 100 healthy subjects ranged from 2–6.8 kPa (Table 2).

**Table 2:** Liver stiffness values (kPa) for the different methods.

*Method*	2D-SWE GE S8	Samsung RS80A SWE	Fibroscan (TE)
Mean LS, kPa	4.5	4.1	4.2
Range	2.9–6.3	2.5–6.8	2.0–6.4
SD	0.8	0.8	1.1
95% CI	4.37–4.67	3.91–4.23	4.0–4.5
CV	0.17	0.21	0.27
CV [range]	0.05–0.28	0.03–0.28	0.04–0.20

Liver stiffness (LS) values (kPa) for 2D-SWE GE, Samsung RS80A and TE. Data are presented as mean with 95% Confidence Interval (CI) and standard deviation (SD), coefficient of variation (CV) and the range of CV for the respective methods.

**Fig 4:**
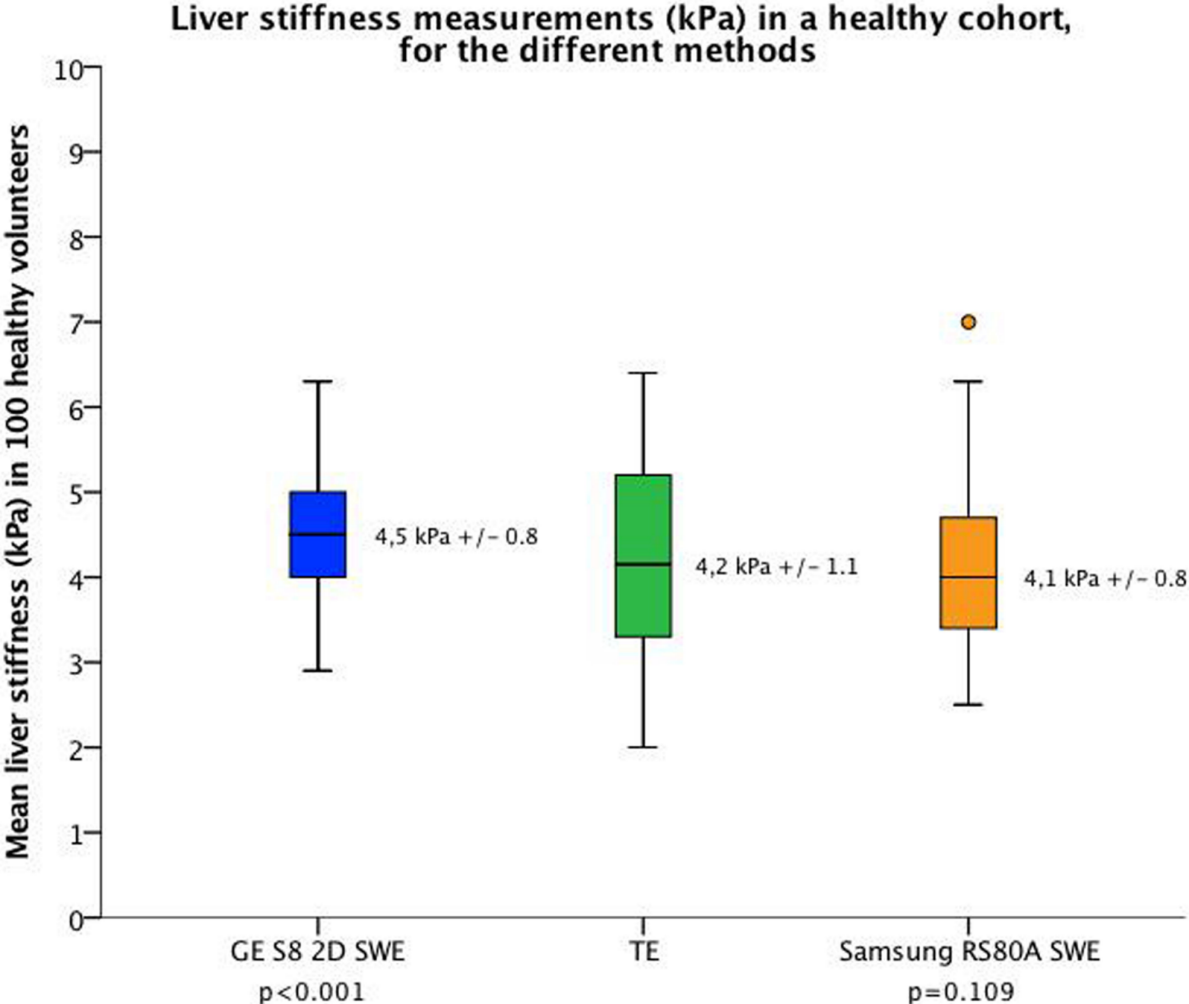
Liver stiffness (kPa) in a healthy cohort for the different methods. This boxplot figure displays the median and the interquartile range for LSM for each system. Whiskers represent the 90% percentile of the measured liver stiffness. The height of the box represents the variability in LSM between the healthy study subjects for each of the following three systems: blue, GE S8 2D-SWE; green, Transient Elastography (TE, Fibroscan) and orange, Samsung RS80A SWE. P-values indicate if there is a significant difference between the novel systems (Samsung RS80A or GE S8) and TE.

LSM-mean by GE S8 2D-SWE was significantly higher compared to LSM-mean by TE (4.5 ± 0.8 kPa vs. 4.2 ± 1.1, respectively, p<0.001) and Samsung RS80A (4.1 ±0.8 kPa, p>0.001), whereas no significant difference was seen between Samsung RS80A and TE (p = 0.11) (Fig 4).

**Fig 5:**
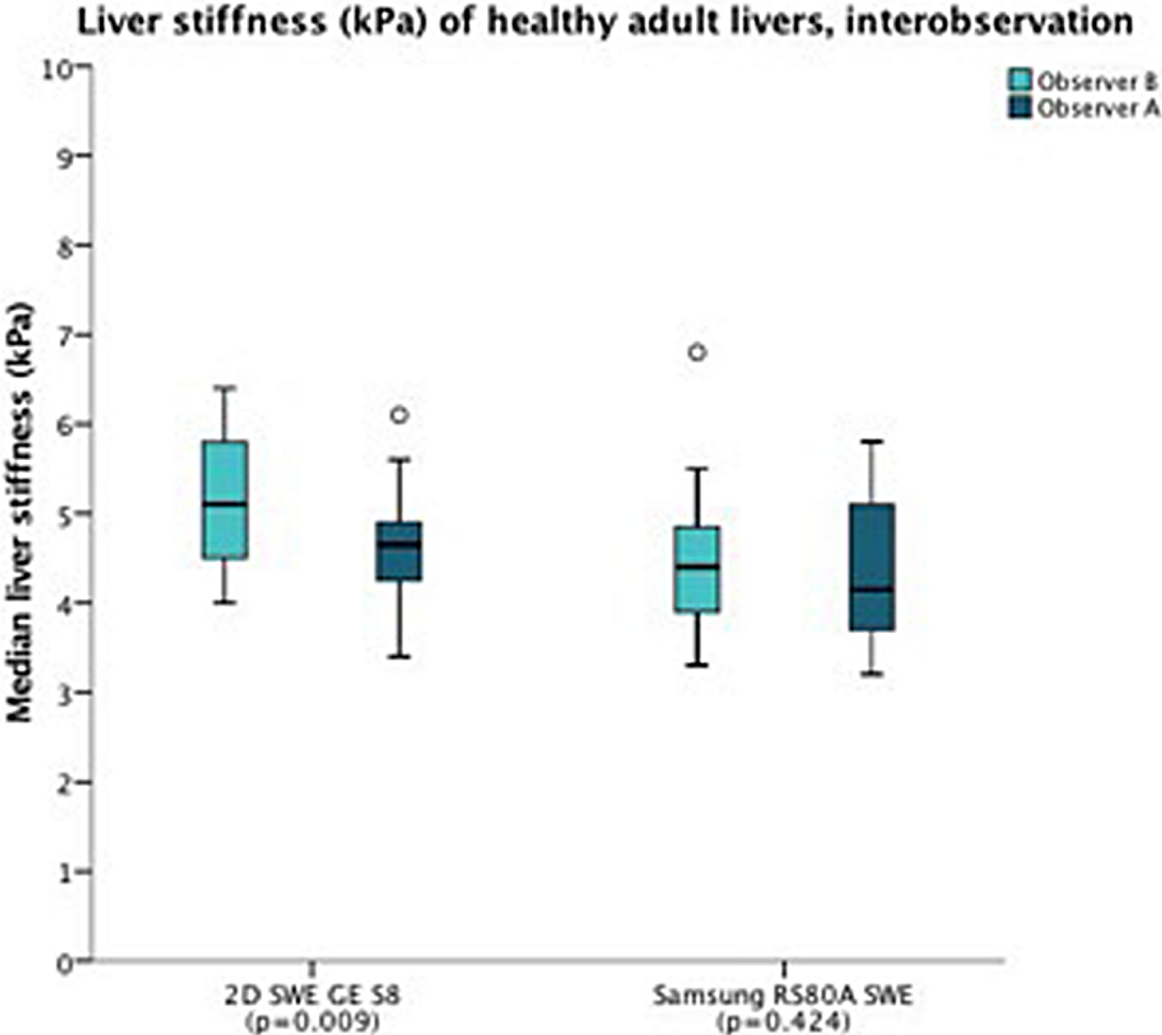
Liver stiffness (kPa) of healthy adult livers, interobservation. The boxplot shows interobservation between observer A (dark blue) and B (light blue). The horizontal axis represents the systems Samsung RS80A SWE and GE S8 2D-SWE and the p-value is given. For boxplot interpretation, we refer to Fig 4.

The coefficient of variation (CV) ranged from 0.03–0.28 for all systems (0.03–0.28 for Samsung RS80A SWE, 0.05–0.28 for GE S8 2D-SWE and 0.04–0.20 for TE). TE had a significantly higher CV than GE S8 2D-SWE (p<0.001) and Samsung RS80A (p = 0.005). Furthermore, between GE S8 2D-SWE and Samsung RS80A we found a small, but significant difference in CV (p = 0.03). Interobserver analysis was performed on 24 randomly selected subjects. No significant differences in LSM-mean between two independent observers (A.M. and A.B.M) was demonstrated for Samsung RS80A SWE (4.4 ± 0.8kPa vs. 4.4 ± 0.8 kPa, respectively, p = 0.42), however, we did find a significant difference between observers for GE S8 2D-SWE (4.5 ± 0.6 kPa vs. 5.1 ± 0.7 kPa, respectively, p = 0.009) (Fig 5).

**Fig 6:**
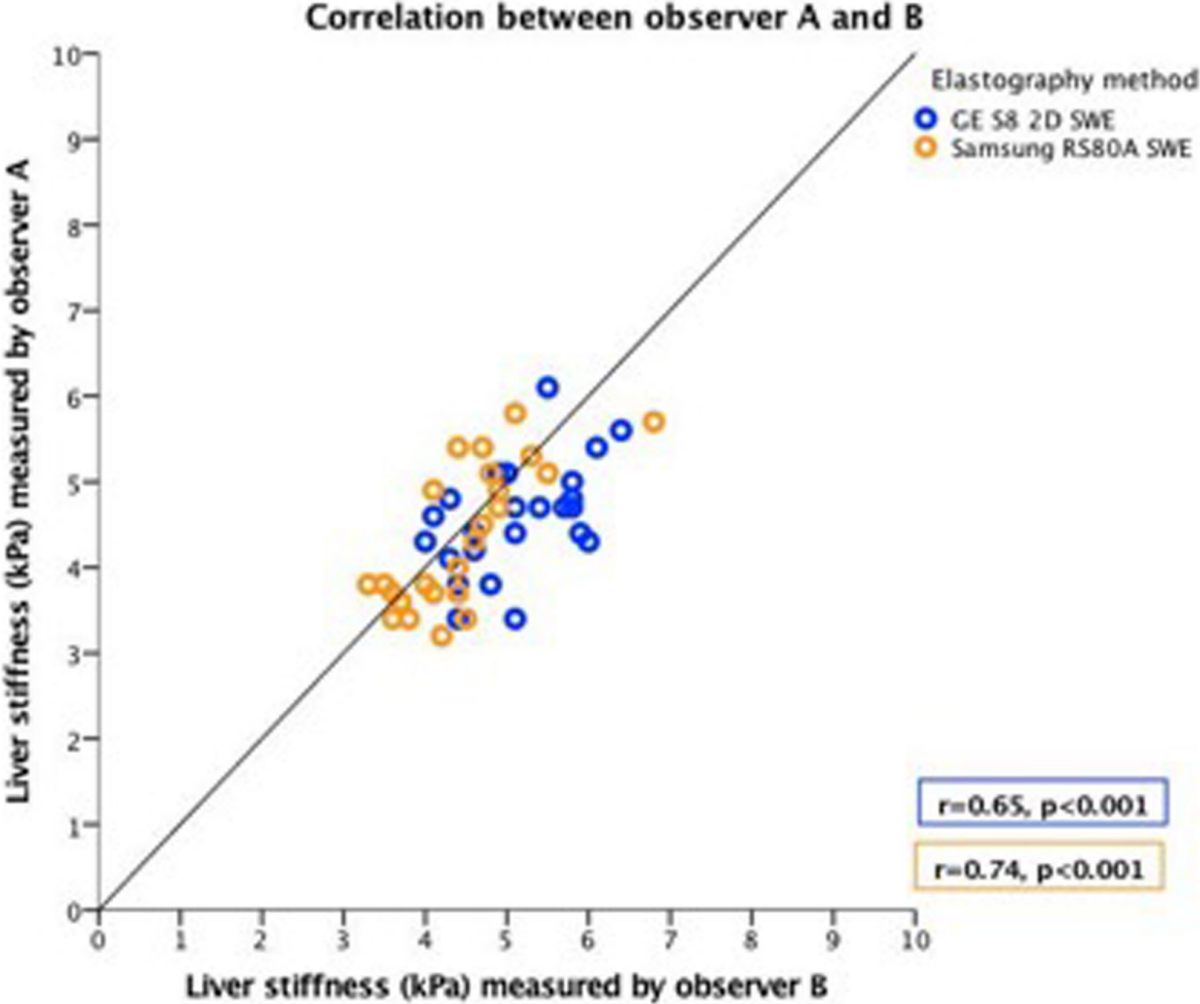
**Correlation between observer A and B.** The horizontal and vertical axes represent measurements by observer B and A, respectively. The unit measured is kilopascals (kPa). The line in the graph represents the line of unity. The Pearson correlation coefficient (r) and significance (p) for each system is given in the lower right corner. For colour representation, we refer to Fig 4.

Interoperator reliability was good for both Samsung RS80A SWE and GE S8 2D-SWE. Pearson’s correlation coefficient between observers was significant for both methods (r = 0.74, p<0.001 vs. r = 0.65, p<0.001, respectively) (Fig 6).

**Fig 7:**
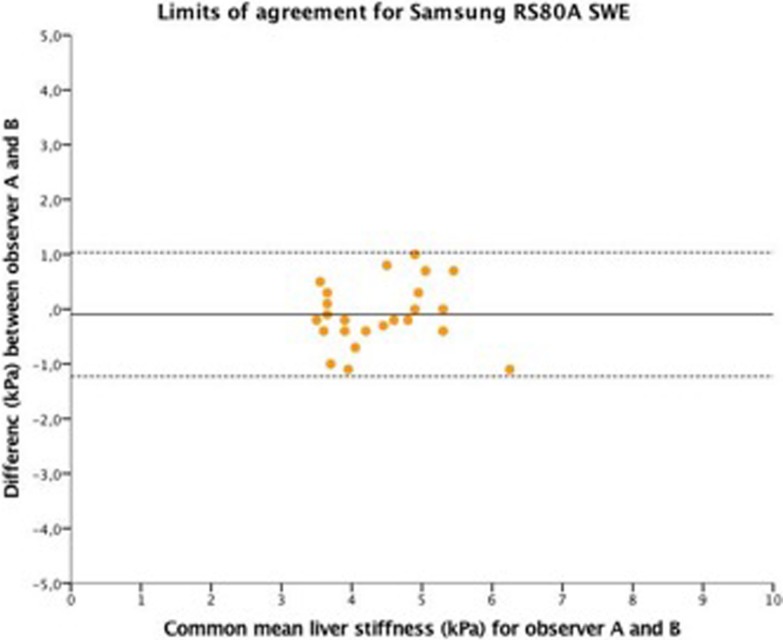
Limits of agreement for Samsung RS80A SWE. The figure presents the limits of agreement for Samsung RS80A. The horizontal axis represents the common mean value of all measurements in both observers for, while the vertical axis represents the difference between individual measurements and this common mean (kPa), displaying the variability of measurements. The black line within each system represents the common mean value, the dotted lines represent the 95% confidence intervals. A mean value close to 0 on the vertical axis means that the two observers apply the measurement scale without bias.

**Fig 8:**
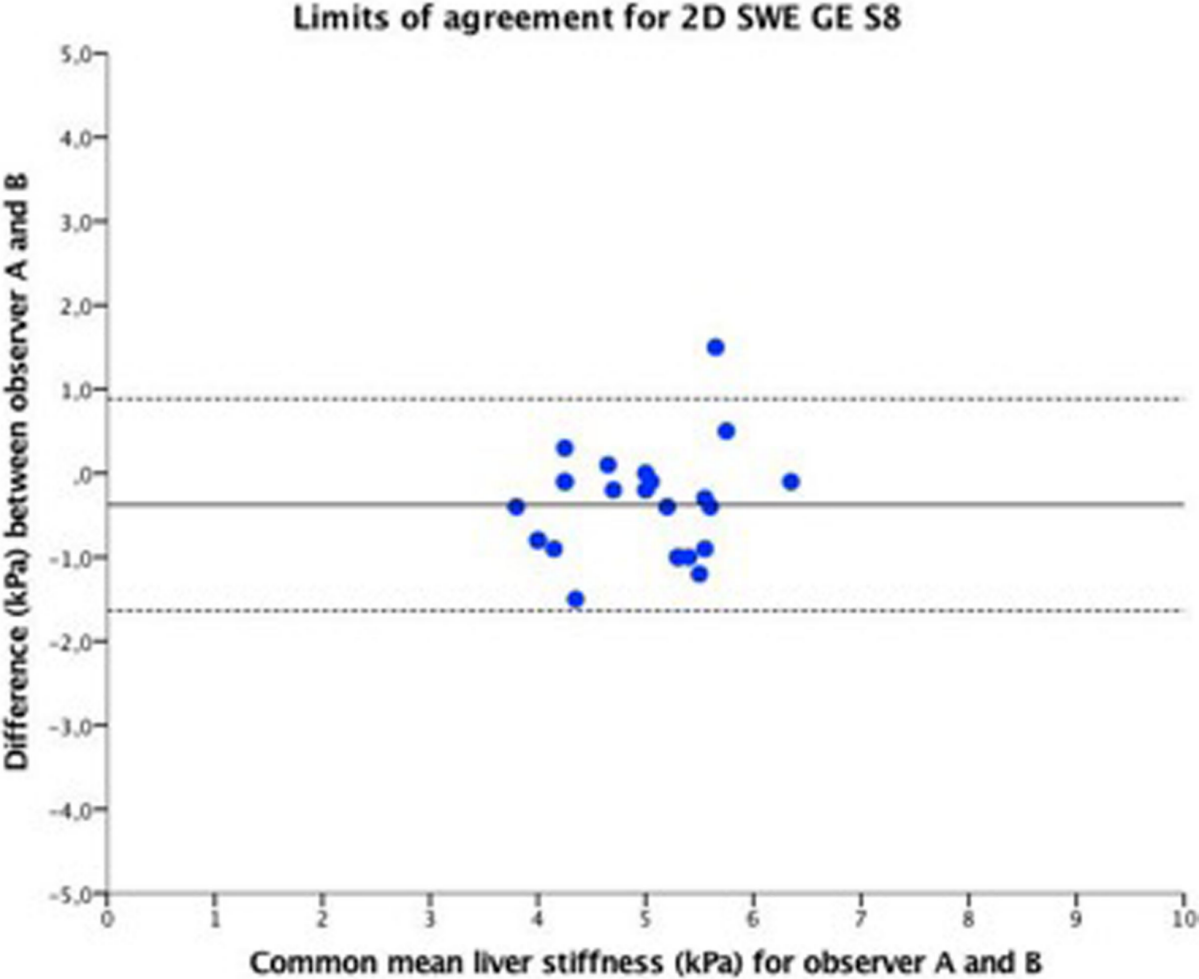
Limits of agreement for 2D-SWE GE S8. Limits of agreement for 2D-SWE GE S8, for legend we refer to Fig 7.

The intraclass correlation coefficient (ICC) was good for both Samsung RS80A and GE S8 2D-SWE (ICC = 0.85 vs. ICC = 0.78, respectively). There was no indication of observer bias for either GE S8 2D-SWE or Samsung RS80A SWE as illustrated by limits of agreement analysis; however, GE S8 2D-SWE showed a trend of a slightly larger deviation of the mean than Samsung RS80A SWE (Figs 7 and 8).

### Difference in liver elasticity by gender, age and BMI

**Fig 9:**
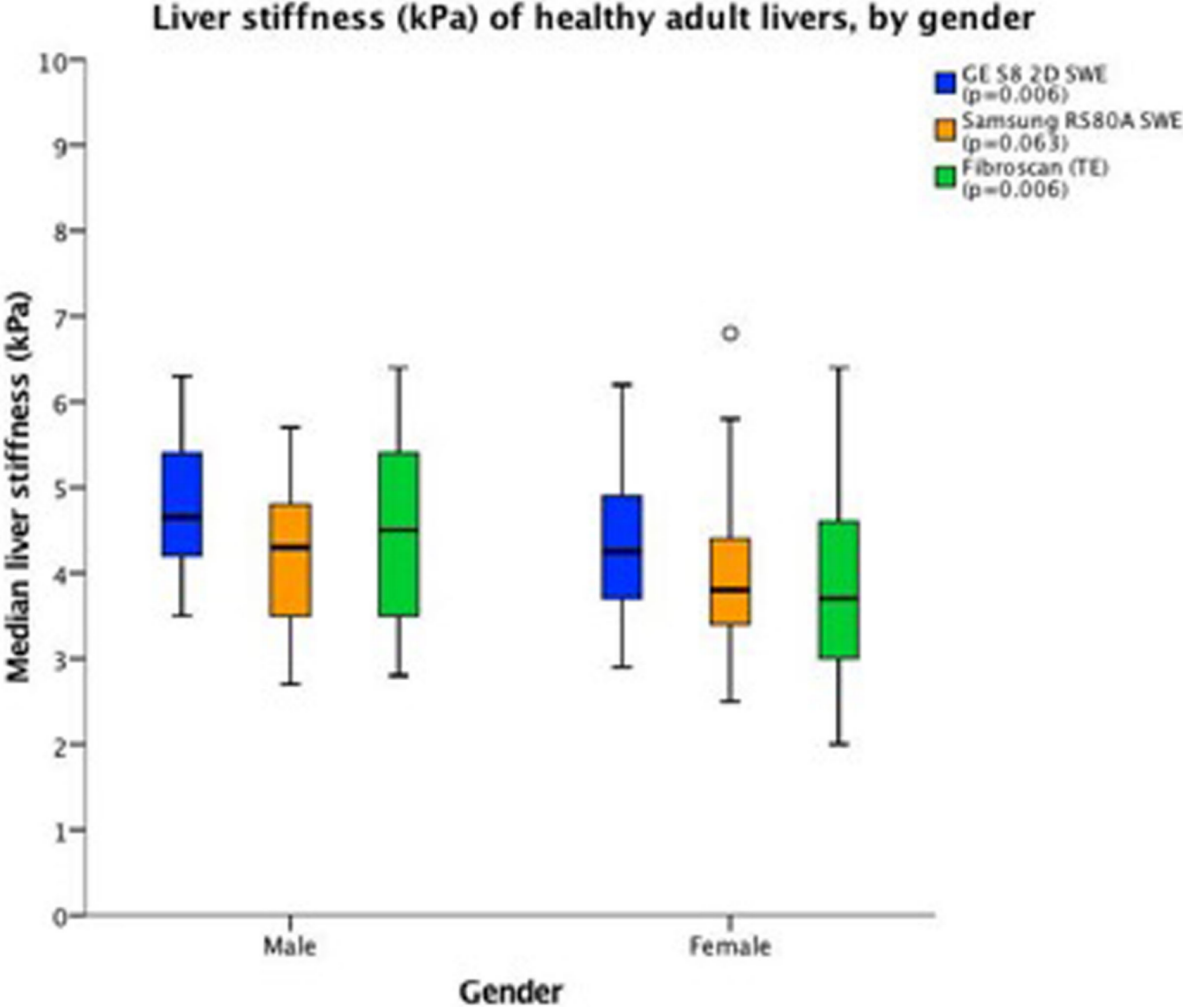
Liver stiffness (kPa) of healthy adult livers, by gender. The boxplot shows the liver stiffness by gender. The horizontal axis represents the gender; males and females. The colour interpretation for each system and the level of significance is given in the upper right corner. For legend interpretation, we refer to Fig 4.

LSM-mean was significantly higher in males compared to females for TE (4.5 ± 1.0 kPa vs. 3.9 ± 1.1 kPa, respectively, p = 0.006) and GE S8 2D-SWE (4.7 ± 0.7 kPa vs. 4.3 ± 0.7 kPa, respectively, p = 0.006). A similar trend for Samsung RS80A SWE did not reach significance (4.2 ± 0.7 kPa vs. 3.9 ± 0.9 kPa, respectively, p = 0.063) (Fig 9, Table 3). In a post hoc analysis of subjects consuming 5 alcohol units or less per week (n = 69) we found significant differences in LSM-mean between males (n = 33) and females (n = 36) for all systems; for GE S8 (4.8 ± 0.7 kPa vs. 4.2 ± 0.8 kPa, p = 0.003), TE (4.7 ± 1.0 kPa vs. 3.8 ± 1.1 kPa, p = 0.001) and Samsung (4.2 ± 0.7 kPa vs. 3.8 ± 0.7 kPa, p = 0.006).

**Table 3:** Liver stiffness values (kPa) of healthy adult livers, by gender.

*Gender*	Female (n = 50)	Male (n = 50)	p-value
**GE S8 2D-SWE**			
Mean ± SD (kPa)	4.3 ± 0.7	4.7 ± 0.7	p = 0.006
95% CI	4.1–4.5	4.5–4.9	
**Samsung RS80A SWE**			
Mean ± SD (kPa)	3.9 ± 0.9	4.2 ± 0.7	p = 0.063
95% CI	3.7–4.2	4.0–4.4	
**Fibroscan (TE)**			
Mean ± SD (kPa)	3.9 ± 1.1	4.5 ± 1.0	p = 0.006
95% CI	3.6–4.2	4.2–4.8	

Liver stiffness values (kPa) for 2D-SWE GE, Samsung RS80A and TE, by gender. Data are presented as mean ± standard deviation with 95% Confidence interval (CI).

None of the systems demonstrated statistical significant difference in LSM across age groups (Table 4).

**Table 4:** Liver stiffness values (kPa) of healthy adult livers, by age group.

*Age group*	20–30 (n = 20)	31–40 (n = 20)	41–50 (n = 20)	51–60 (n = 20)	61–70 (n = 20)	p-value
**GE S8 2D-SWE**						
Mean ± SD (kPa)	4.5 ± 0.9	4.7 ± 0.8	4.5 ± 0.7	4.4 ± 0.7	4.5 ± 0.7	p = 0.843
95% CI	4.1–4.9	4.3–5.1	4.1–4.8	4.1–4.8	4.2–4.9	
**Samsung RS80A SWE**						
Mean ± SD (kPa)	4.3 ± 0.9	4.2 ± 0.8	4.0 ± 0.8	4.1 ± 0.6	3.9 ± 1.0	p = 0.630
95% CI	3.9–4.7	3.8–4.5	3.6–4.4	3.8–4.3	3.4–4.4	
**Fibroscan (TE)**						
Mean ± SD (kPa)	4.4 ± 1.1	4.3 ± 1.3	4.2 ± 1.0	4.2 ± 1.1	4.1 ± 1.1	p = 0.630
95% CI	3.9–4.9	3.7–5	3.7–4.7	3.7–4.7	3.6–4.7	

Liver stiffness values (kPa) for 2D-SWE GE, Samsung RS80A and TE, by age group. Data are presented as mean ± standard deviation with 95% Confidence interval (CI).

**Fig 10:**
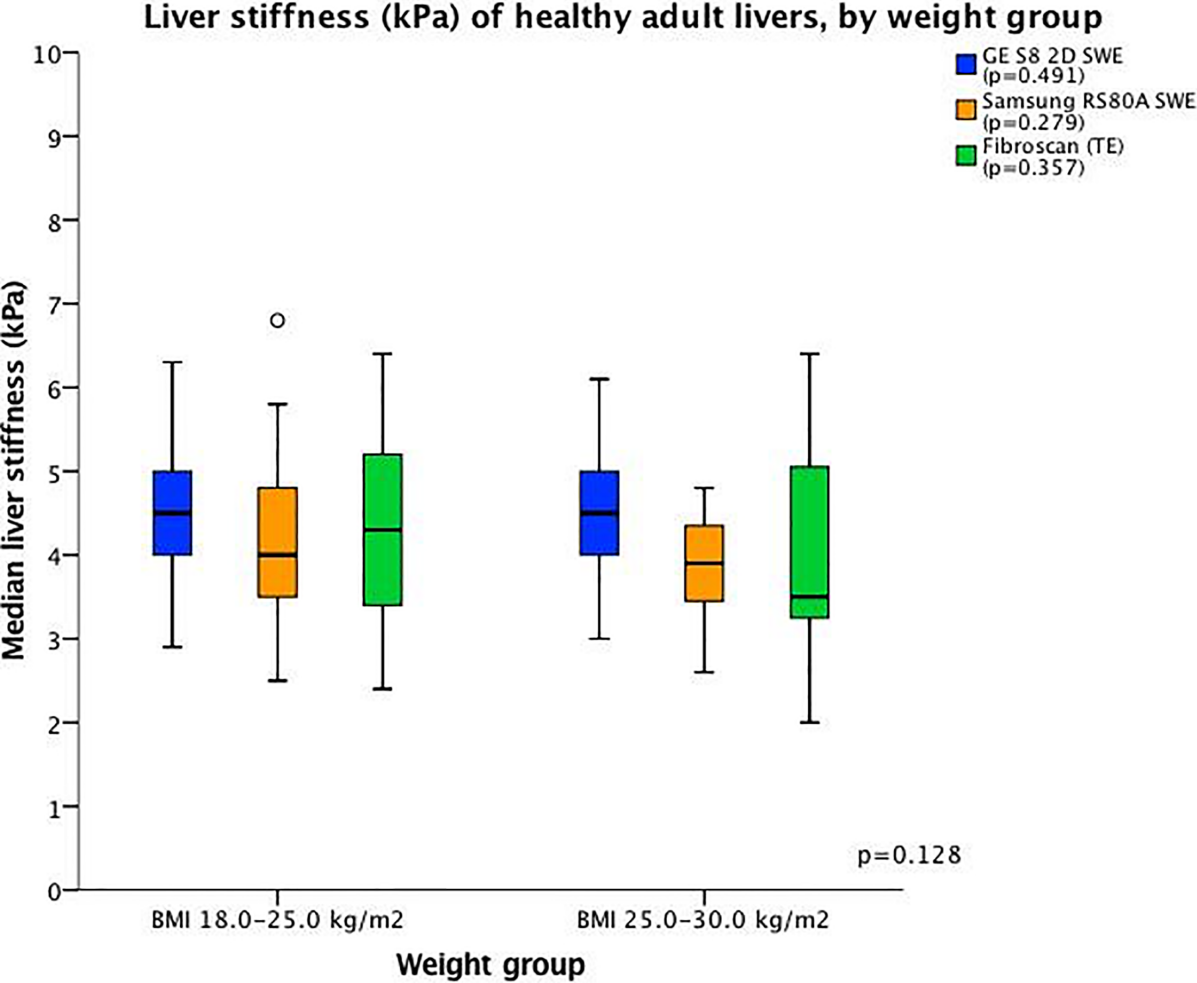
Liver stiffness (kPa) of healthy adult livers, by weight group. The boxplot shows the liver stiffness by weight group. The horizontal axis represents the BMI group. The colour interpretation for each system and the level of significance is given in the upper right corner. For boxplot interpretation, we refer to Fig 4.

LSM-mean showed no significant difference between subjects with BMI 25–30 kg/m^2^ and BMI 18.0–25.0 kg/m^2^ for any individual system (GE S8 2D-SWE (4.5 ± 0.8 kPa vs. 4.4 ± 0.8 kPa, respectively, p = 0.49), TE (4.3 ± 1.1 kPa vs. 4.1 ± 1.1 kPa, respectively, p = 0.36), Samsung RS80A SWE (4.1 ± 0.9 kPa vs. 3.9 ± 0.6 kPa, respectively, p = 0.28) or all systems combined (4.1 ± 0.9 kPa vs. 4.3 ± 0.9 kPa, p = 0.128) (Fig 10).

### Difference in variability and reproducibility of LSM when using 5 measurements instead of 10

**Fig 11:**
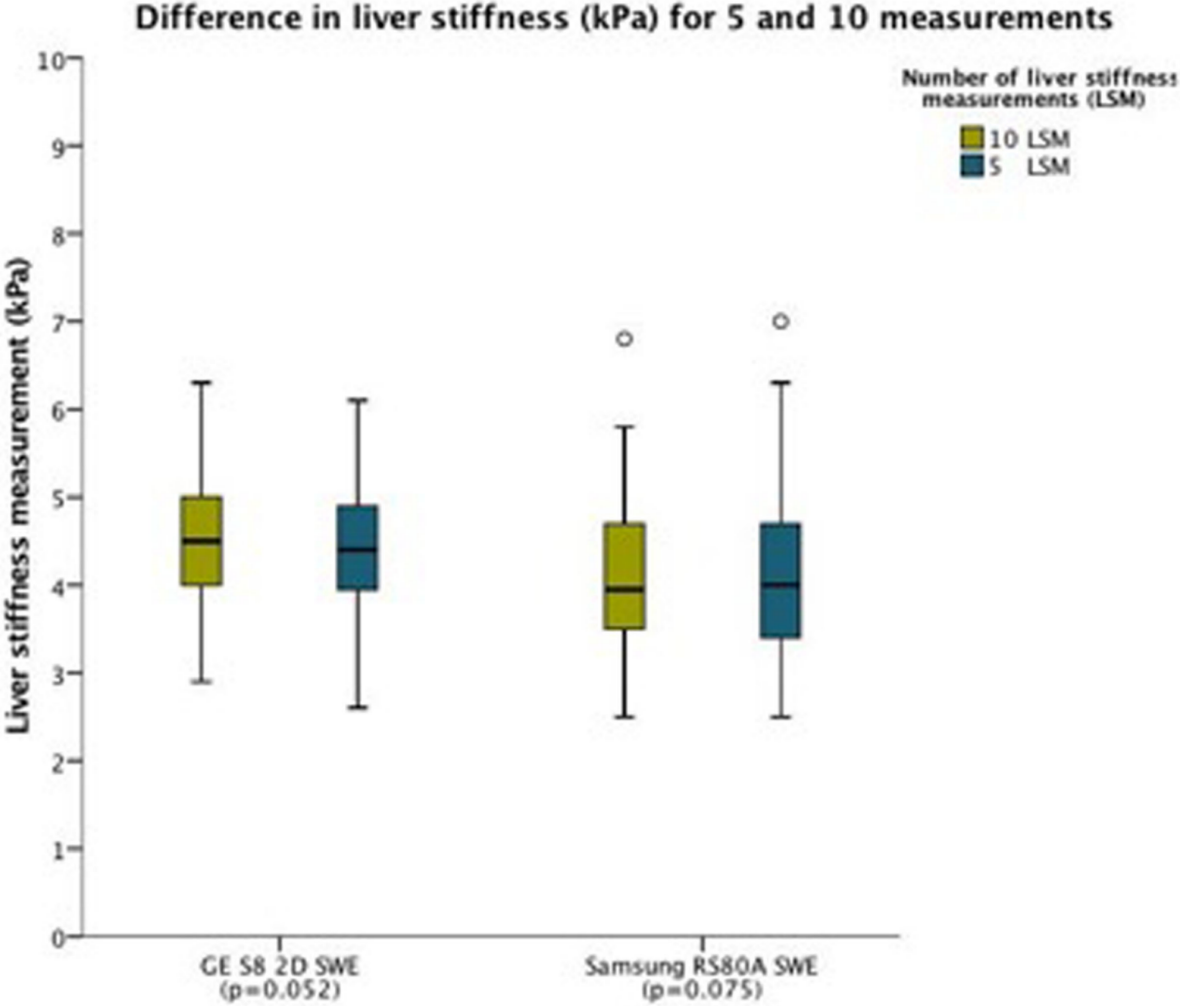
Difference in liver stiffness (kPa) for 5 and 10 measurements. The boxplots show difference in liver stiffness for 5 and 10 measurements. The horizontal axis represents the systems, and the vertical axis the liver stiffness measured. The colour interpretation for 5 and 10 measurements (green and blue, respectively) is given in the upper right corner. For boxplot interpretation, we refer to Fig 4.

There was no significant difference in LSM-mean using 5 or 10 measurements for the ultrasound based SWE methods (GE S8 2D-SWE 4.4 ± 0.66 kPa vs. 4.5 ± 0.76 kPa, respectively, p = 0.05; and Samsung RS80A SWE: 4.1 ± 0.86 kPa vs. 4.1 ± 0.81 kPa, respectively, p = 0.08) (Fig 11).

## Discussion

To the best of our knowledge, this is the first study to investigate normal LSM values by two new elastography techniques (Samsung RS80A SWE and GE S8 2D-SWE) compared head-to-head and with TE as reference, in a healthy cohort. The comprehensive exclusion of liver disease as well as the direct comparison to TE as a reference standard represent strengths of our study. Data regarding normal values in liver stiffness for each of the new elastography techniques are needed to establish standardized reference bases [12], which are pivotal for clinical implementation of novel elastography systems as reliable methods for diagnostics, staging and assessment of disease progression in chronic liver diseases.

We found a mean LSM of 4.3 kPa ± 0.8 across the two methods, confirming that on average LSM for 2D-SWE GE S8 (4.5 ± 0.8 kPa) and Samsung RS80A (4.1 ±0.8 kPa) were in the same range as other elastography systems [13–15]. In this head-to-head comparison between elastography systems, 2D-SWE GE S8 demonstrated slightly higher values than both Samsung RS80A and TE, while measurements made with Samsung RS80A were not significantly different from the reference method. There was also a small, but significant difference in the coefficient of variation between the two novel methods. Previous studies have shown similar results for 2D-SWE from Aixplorer [16, 17]. Our results confirm that LSM levels are significantly different depending on the method applied. We found differences both between SWE methods and TE as the reference method, and between the two different SWE systems. In clinical practice LSM greater than 6.8–7.6 kPa indicates a higher probability of significant fibrosis (F ≥2) on liver biopsy; however, the EASL clinical practice guidelines state that cut-off values vary considerably and ranging 5.2–9.6 kPa for different systems. For predicting cirrhosis (F4), the optimal cut-off ranges from 11 to 15 kPa [18]. In that context, a net difference of 0.3 kPa is probably too small to represent a clinically significant difference, however, it underscores the need to compare methods also in fibrotic livers, where the differences may be more expressed, as we know that variability increases with higher liver stiffness [19].

Both methods showed good interobserver reliability and intraclass correlation. Similarly, previous studies have shown excellent interobserver agreement ranging from r = 0.80–0.97 for pSWE methods [20, 21]. However, we observed a significant difference in LS measurements between the two observers for 2D-SWE GE S8 but not for Samsung RS80A. One possible explanation for this discrepancy may be that 2D-SWE allows the examiner to place the measurement ROI within the elastogram and avoid incongruent signals, while Samsung SWE performs several automated SWE speed measurements within the elasticity measurement area without visualisation of the stiffness. The 2D-SWE method is slightly more user dependent and may acquire a longer learning curve. Previous studies on 2D-SWE measurements of liver elasticity have demonstrated a learning curve for this method, but with similar reproducibility [16, 22]. Evaluating the intra- and interobserver agreement for 2D-SWE from GE and SWE from Samsung, we demonstrated a good interobserver and better intraobserver agreement for both systems compared to the results reported for Aixplorer 2D-SWE (Figs 7 and 8).

We found a significantly higher LSM in adult male subjects for TE and GE S8 2D-SWE, whereas a similar trend for Samsung RS80A SWE did not reach significance. This is an important finding, indicating that it may be necessary to define separate cut-off values for normal liver and possibly also for levels of liver fibrosis for male and female patients. Previous studies have shown inconsistent results regarding the effect of gender on LSM [23, 24]. Using pSWE, Ling et al. demonstrated that males had 8% higher LSM than females; however, the study had more than twice as many female participants compared to male participants [14]. In contrast, using ARFI, one study found no significant difference between genders in 137 subjects [25] in line with our results for Samsung RS80A. Two studies conducting reliable LSM with TE in 1190 subjects over 45 years, and in 746 healthy subjects, found that male gender was associated with higher liver stiffness [26, 27] and our results for TE confirmed this. Using Aixplorer 2D-SWE from Supersonic Imagine, it has been suggested that males may have higher LSM than females [13]. A study of LSM in healthy children, using the same system, did not demonstrate significant difference between genders [28]. The lack of significant gender difference for LSM in healthy liver tissue for Samsung SWE in the present study may be due to different technology and signal processing compared to the two other scanners. Despite that we found a significant difference between genders for all systems in our post hoc analysis (n = 69), the study may be underpowered considering the observed SD of 0.8 kPa for the Samsung SWE compared to our power estimation anticipating 0.5 kPa as SD. Furthermore, different hormone levels have been proposed as an explanation of LSM differences between genders, and should be investigated further in *in vivo* studies [29].

In our study, LSMs were not significantly affected by age or BMI. Multiple studies have addressed age as a variable of influencing liver stiffness in normal subjects, and the results have been inconsistent, reporting no difference across age groups [14], higher LSM in older [26] or younger [27] age. We did not demonstrate significant differences in LSM between the five age groups for any of the methods. Possibly, analyses of the effect of age on LSM is confounded by other factors such as steatosis and heart failure that are more prevalent in older populations. One study investigating GE E9 Logic 2D-SWE in healthy subjects reported an LSM-mean of 5.1 kPa ± 1.3, with higher LSM values compared to TE, similar to our findings [17]. In contrast to our results, they reported that age over 40 years was associated with higher LSM, but did not find significant difference in LSM between genders (21 males and 58 females). In the present study, we included only healthy volunteers, carefully interviewed all subjects regarding alcohol consumption, and performed full biochemical analyses and B-mode ultrasound examination of all in contrast to some other studies [12], and in our view, less strict inclusion criteria and missing data regarding liver enzymes in the other study may contribute to these differences. Higher LSM values have been reported in healthy subjects with low BMI (<18.5 kg/m^2^) as well as in obese subjects compared with normal-weight subjects [30]. We did not demonstrate a difference in LSM between subjects with BMI 18.0–25.0 kg/m^2^ compared to BMI 25–30 kg/m^2^; however, obese patients with BMI>30 were not included in this study. Normal values for LS in underweight and obese subjects, as well as technical feasibility of Samsung RS80A SWE and GE S8 2D-SWE, should be further investigated and established.

There is an ongoing discussion of the minimum number of measurements needed when acquiring LSM with SWE. The EFSUMB guidelines recommend at least 10 measurements for pSWE and TE, and a minimum of 3 measurements when using 2D-SWE, to obtain consistent results [31, 32]. One study reported excellent intraobserver reproducibility based on 6 measurements, concluding that the optimal minimum number of measurements with 2D-SWE was 6 [15]. For pSWE (ARFI), one study concluded that 10 measurements instead of 5 should be performed to obtain a reliable estimation [33]. To the best of our knowledge there are no studies that have directly investigated the difference in mean LS between 5 and 10 separate measurements for several ultrasound SWE methods. Our results did not show significant difference in median LS for 5 versus 10 measurements. This suggests that a reliable median LSM can be obtained with fewer measurements than ten for both 2D-SWE GE S8 and Samsung RS80A in healthy livers. This is important as it indicates that adequate measurements can be made by fewer repetitions and in less time, however our results in healthy livers may not apply in patients with higher degree of liver fibrosis where measurement variability may be higher.

The main limitation of the study is the lack of liver biopsies as a reference method, which is not ethically feasible in a healthy group. Our study design included 100 healthy participants, excluding unknown liver disease by imaging, blood tests and anamnesis.

## Conclusion

All methods were successfully applied in our cohort of 100 healthy subjects. The mean of median LSM for the two new elastography methods (GE S8 and Samsung RS80A) showed a slight difference. Our study shows a significantly higher liver stiffness in males compared to females, however we found no significant difference in LS between BMI groups 18–30 kg/m^2^ or between the age groups 20–70 years. Furthermore, our findings indicate that five acquisitions are sufficient to obtain a reliable LSM using Samsung RS80A or GE S8 2D-SWE in healthy subjects.
